# An improved approach of totally visceral sac separation (TVS) for incisional hernia compared with laparoscopic intraperitoneal onlay mesh plus repair (IPOM plus)

**DOI:** 10.1038/s41598-023-45192-2

**Published:** 2023-10-21

**Authors:** Bo Zhuang, Lushan Zheng, Shian Yu, Gang Li

**Affiliations:** 1 Department of General Surgery, Jinhua Hospital, Zhejiang University School of Medicine, Jinhua, China; 2 College of Mathematical Medicine, Zhejiang Normal University, Jinhua, China

**Keywords:** Diseases, Medical research

## Abstract

Endoscopic techniques have been widely used in ventral hernia surgery. Totally visceral sac separation (TVS) is a new concept proposed for hernia repair in recent years. The aim of this study was to contrast the postoperative results of TVS with the widely used method of Laparoscopic intraperitoneal onlay mesh plus repair (IPOM plus) for incisional hernias. The retrospective comparison analysis of 38 IPOM plus and 34 TVS was conducted during the time period between December 2019 and June 2022. For both two groups, baseline characteristics, surgical records, postoperative information, and quality of life outcomes utilizing the Carolina’s Comfort Scale were collected and analyzed. There were no differences between the methods of TVS and IPOM plus among the baseline characteristics. It showed the operative time in TVS group with the mean time of 213.4 min was significantly longer than that in IPOM plus group with the mean time of 182.9 min (*P* = 0.010). The postoperative length of stay in TVS group was 6.2 days, which was significantly shorter than IPOM plus group with the mean time of 4.8 days (*P* = 0.011). The medical expenses was significantly smaller in TVS group than that in IPOM plus group (*P* < 0.001). The quality of life scores of TVS were significant better than IPOM plus at one week, one month and six months. Besides, both TVS and IPOM plus have very few complications. TVS approach for incisional hernias is secure, effective, and valuable. It has shorter postoperative length of stay, higher quality of life, longer operative time, smaller medical expenses, and approximate complications compared with IPOM plus procedure. Our results have a greater contribution to the application and popularization of TVS technique.

## Introduction

One of the most frequent surgical conditions treated by surgeons is ventral hernia. LeBlanc and Booth have firstly published intraabdominal positioning of the prosthetic mesh (IPOM) with non-adhesive coating, which is regarded as a milestone in ventral hernia surgery due to the advancements of minimally invasive technique^1^. Then the IPOM technique the laparoscopic closure of the defect (IPOM plus) had been reported by Franklin^2^. It has already received extensive acceptance due to its little impact on the stability of the abdominal wall and great reproducibility. The primary disadvantage of it is, however, the increased risk of intestinal injury and potential for long-term problems from intraperitoneal mesh placement. Despite the remarkable developments of industry's and mesh technology, the significant risks persisted. Furthermore, IPOM plus do not have any advantages in acute or chronic pain and quality of life^3^.

In 2012, the extended view totally extraperitoneal (e-TEP) repair for inguinal hernia was first reported by Jorge Daes and adopted for ventral hernia by Belyansky et al. later^4,5^. The non-composite mesh is positioned in the retromuscular plane without fixation. Although it reduces the risk of visceral injury bought by the intraperitoneal mesh, the posterior sheath of retromuscular (PSR) is broken by the technique of Crossover and transversus abdominis release (TAR).

On the basis of e-TEP, many scholars have put forward the new concepts of totally visceral sac separation (TVS)^6–8^. Although there is some controversy, the concept of TVS involves that the whole abdominal wall could be regarded as a physiological and functional entity which is composed of multiple anatomical structures and planes. Surgical approaches or techniques can break down the boundaries of the abdominal wall, including the linea alba separating retromuscular space; the linea semilunaris separating retromuscular space from extraperitoneal space; and scars from previous abdominal surgeries separating the originally connected tissue space. If separated spaces are connected in preperitoneal plane, an ample preperitoneal space is established without the technique of Crossover and TAR. And then a sufficient space can be built to accommodate to large polypropylene mesh without fixation. It is as little as possible to affect the biomechanical stability of the abdominal wall. Entering into the preperitoneal space of the abdominal wall without utilizing the Crossover and TAR techniques differs from the e-TEP approach^9^. And the major differences between the TVS approach and IPOM plus are the choice of the preperitoneal plane for non composite mesh.

The purpose of this study is to evaluate the benefits and drawbacks of the latest method of TVS and the earliest method of IPOM plus for incisional hernias from multiple dimensions, such as operative time, postoperative length of stay, complications, and quality of life.

## Materials and methods

### Patients and data collection

The department of endoscopic surgery performed reviews on 34 patients who underwent TVS repairs and 38 patients who underwent laparoscopic IPOM plus procedures for incisional hernia at the tertiary care center between December 2019 and June 2022. There are no variations in the patients of co-morbidities (including diabetes, chronic obstructive pulmonary disease (COPD), cardio-vascular, active smoking, immunosuppression), and the preoperative CCS scores. Baseline data were collected and compared, including sex, age, and BMI. And each one of these 72 patients had been classified using the EHS system^10^. The study had also collected the defect's size and area by physical examination and using preoperative CT for each patient. Operative time, postoperative length of stay, medical expenses, readmission rates, and complications, were extracted from the surgical records. Quality of life were followed up and collected utilizing the Carolina’s Comfort Scale (CCS) at post-discharge one week, one month, and six months^5^. CCS is a validated hernia-specific survey that utilizes a 0–5 scale to assess pain, mesh sensation, and limitations in mobility. A significant score on any of the three measured CCS parameters had been previously defined as ≥ 2 out of 5 and was used for our analysis^3^. The CCS scores were recorded by an independent blinded observer. The analysis also took the postoperative length of stay and readmission rates into account. The collection and review of retrospective data from previous medical records were authorized by the local ethic committee and institutional review board. The procedure was approved by the medical ethics committee of Jinhua Hospital in compliance with the Helsinki Declaration’s standards. The corresponding ethical approval code is 2019(191). All methods were carried out in accordance with relevant guidelines and regulations, and informed consent was obtained from all subjects and/or their legal guardian(s).

### Preoperative workup

Patients with multiple abdominal wall defects, active infections or life-threatening conditions were not eligible to participate in the study. A detailed medical history, physical exam, blood tests, and an abdominal CT should all be included in the study. Each patient received antimicrobial prophylaxis prior to surgery. Regularly placed Foley catheters were used for the surgery site's cleaning and general anesthesia.

### TVS synopsis

**Figure 1:**
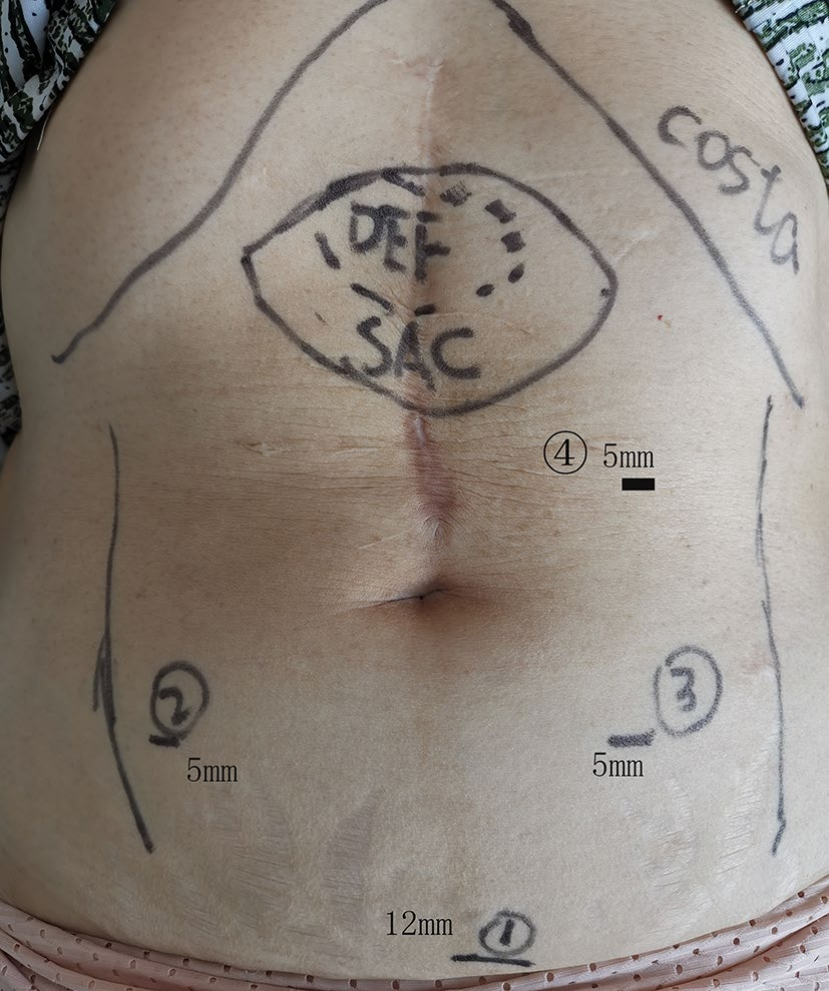
Illustrate the preoperative planning of the trocar layout for epigastric defect in the TVS approach.

**Figure 2:**
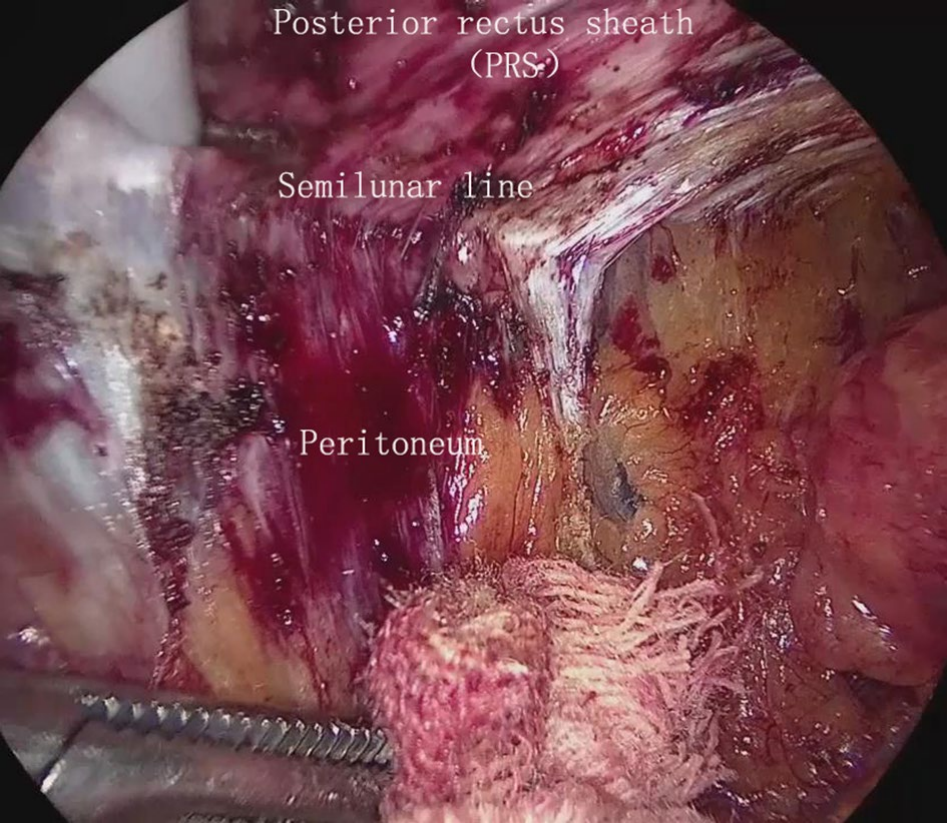
Entry into the preperitoneal space of the lateral abdominal wall without TAR in the TVS approach.

**Figure 3:**
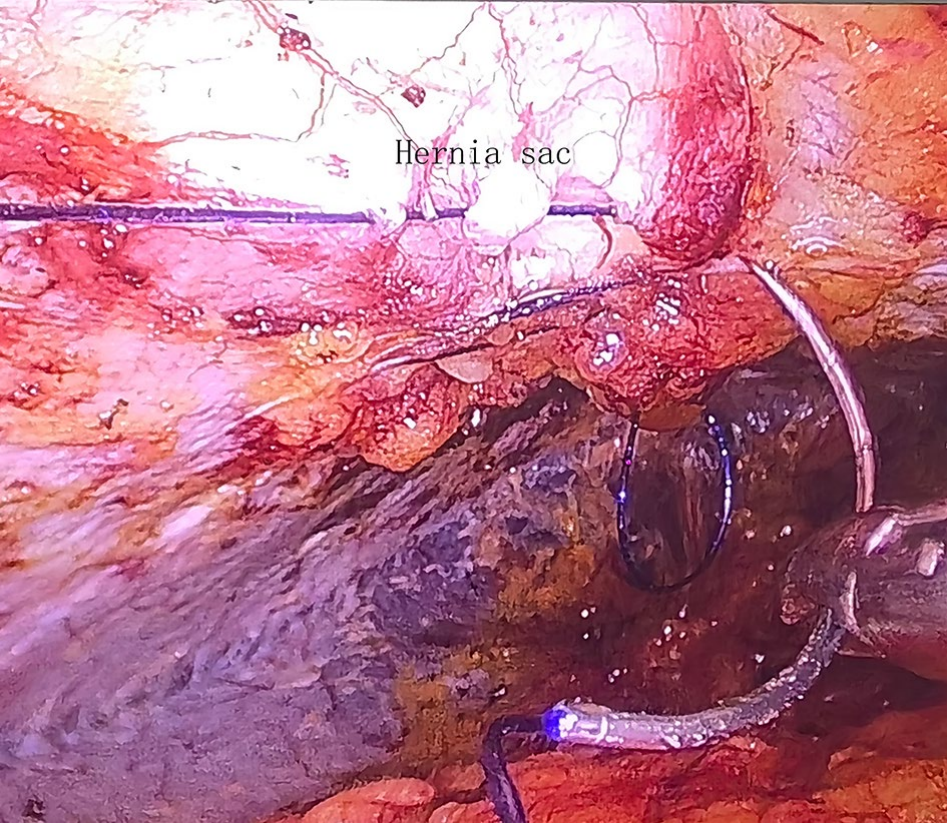
The stereoscopic suture to eliminate the hernia sac in the TVS approach.

The preoperative planning of the trocar layout is the initial step in the TVS approach. The size and location of the defect should be taken into account when putting the trocars in addition to following the basic principles for standard endoscopic surgery. Figure 1 showed the trocar placement and incision sites. Firstly, the optic port in the preperitoneal space is usually established in the lower abdomen. We are employing the established methodology previously disclosed for the TVS approach^5^ or the visual method^11^. Secondly, it is carefully avoided to cause any damage to the inferior epigastric veins. Bilaterally, the 5-mm functioning ports are inserted. The surgeon positions themselves between the patient's legs after creating the initial gap and ports. Usually, one hand is used to pull the peritoneum gently and persistently downward during the separation procedure, and the other hand is used to prepare an electric hook for blunt dissection or directed cautery. Depending on which side is dissected, either hand can grip the electric hook alternately. The peritoneum can be compressed by inserting a small piece of gauze and holding it in place with non-invasive forceps. This aids in easing peritoneal pressure and lowers the possibility of peritoneal rips during retraction. Thirdly, The incision hernia sac could be opened or dissected with the surrounding preperitoneal area about 5 cm. Figure 2 showed the preperitoneal space could be established without the technique of TAR. Fourthly, the posterior defect can be closed after detaching enough peritoneum. The anterior defect is then closed shut using continuous knotless barbed sutures (Sxpp1A406/USA, wound closure device 1–0 by Ethicon LLC). We always perform stereoscopic suture, which fixes the hernia sac to the anterior defect, in order to eliminate the hernia sac and reduce the incidence of postoperative seroma (Fig. 3). Finally, after the preperitoneal space has been established, the medium weight polypropylene mesh is then placed over the peritoneum without fixing it. The surgery process of TVS for hernia in M3 can be seen in the video (TVS for hernia M3).

### IPOM plus synopsis

**Figure 4:**
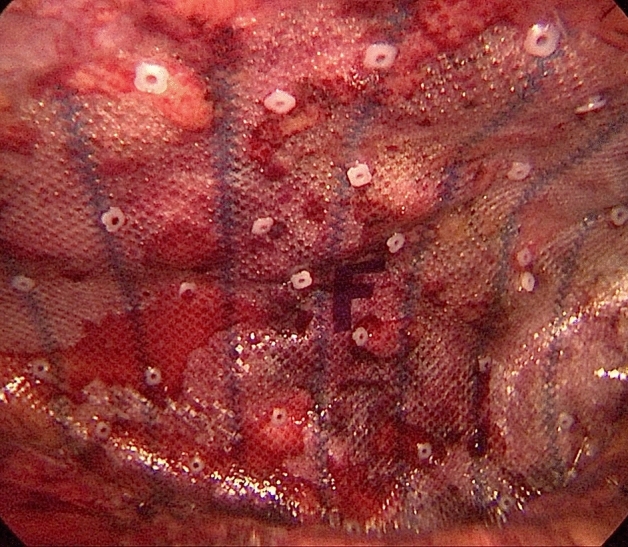
The composite mesh was attached to the abdominal wall.

The control group had undergone the IPOM plus process. Three trocars are typically inserted after a 12 mmHg pneumoperitoneum has been established using a Veress needle or Hasson technique. Sharp and blunt dissection are used to reduce the hernia's contents. Operator should then close the defect. The multi-point approach is used to attach composite mesh to the abdominal wall while maintaining a low pneumoperitoneum (8–10 mmHg) and covering the defect by at least 5 cm on both sides (Fig. 4).

### Statistical analysis

We conducted continuous bivariate analysis using Student's t test with the statistical program IBM SPSS Statistics v27.0. For small sample sizes, the Chi-squared test or Fisher's exact were used to compare continuous nonparametric data. Statistical significance was considered if the p value was less than 0.05.

## Results

### Baseline characteristics

There were 72 patients in total, of which 38 (18 males/20 females) laparoscopic IPOM plus repairs and 34 (11 males/23 females) TVS repairs were included for the further analysis. As shown in Table 1, there are no significant statistical differences in the patients' age, sex, BMI and defect area between these two groups. Besides, the detailed EHS classification and co-morbidities have no differences either. The results are shown in greater detail below.

**Table 1:** Baseline characteristics.

Variable	TVS—34	IPOM—38	*P*
Age (Mean ± SD; years)	58.9 ± 16.7	63.9 ± 11.3	> 0.05
Sex (male/female)	11/23	18/20	—
BMI (mean ± SD)	23.9 ± 3.6	25.1 ± 2.7	> 0.05
Defect area (mean ± SD; cm^2^)	28.2 ± 21.2	33.0 ± 25.7	> 0.05
EHS classification (*N*)			
M1	0	0	
M2	0	1	
M3	19	27	
M4	5	2	
M5	2	0	
L1	0	0	
L2	7	5	
L3	1	2	
L4	0	1	
W1	21	25	
W2	12	11	
W3	1	2	
R	1	1	
Co-morbidity (*N*)			
Diabetes	4	5	
COPD	3	1	
Cardio-vascular	2	3	
Active smoking (*N*)	8	10	
Immunosuppression (*N*)	0	1	

*N* means the absolute number, *SD* refers to the standard deviation, *BMI* refers to body mass index, *EHS* refers to European hernia society, *COPD* refers to chronic obstructive pulmonary disease.

#### Operative and perioperative item

There were significantly statistical differences at operative time, postoperative length of stay, medical expenses, and quality of life, and no statistical differences at readmission and complications between TVS group and the IPOM plus group. The operative and perioperative data are shown in Table 2. The average operating time was 213.3 min for TVS group and 183.9 min for IPOM plus group, respectively. In TVS group, drains were usually removed on the second postoperative day. Thus, the mean postoperative length of stay was significantly shorter in TVS group (4.8 ± 2.0 days) than in IPOM plus group (6.2 ± 2.5 days). The medical expenses in TVS group (1890.3 ± 401.8 USD) were also significantly less than that in IPOM plus group (3302.8 ± 552.3 USD). And the Table 2 also showed the mesh area in TVS group is small than that of IPOM group (< 0.05).

**Table 2:** Operative and perioperative data.

Variable	TVS-34	IPOM-38	*P*
Operative time (mean ± SD; min)	213.4 ± 54.8	182.9 ± 42.4	0.010
PLOS(mean ± SD; days)	4.8 ± 2.0	6.2 ± 2.5	0.011
Medical expenses(mean ± SD; USD)	1890.3 ± 401.8	3302.8 ± 552.3	< 0.001
Readmission(N)	0	0	–
Mesh area (mean ± SD; cm^2^)	147.9 ± 27.39	167.4 ± 22.4	< 0.05

*N* means absolute number, *SD* refers to standard deviation, *NS* refers to non-significant, *PLOS* refers to Postoperative length of stay.

#### Complications

The complications are shown in Table 3. One recurrence at 6 months, one symptomatic seroma, and one hematoma occurred in the TVS group after surgery, and one surgical site infection (SSI), one symptomatic seroma, and one urinary tract infection happened in IPOM plus group. All complications were managed conservatively. There were no reinterventions and perioperative mortality in the 72 samples.

**Table 3:** Complications.

Variable (N)	TVS-34	IPOM-38
Intraoperative complications	0	0
SSI	0	1
Symptomatic seroma	1	1
Hematoma	1	0
Pneumonia	0	0
UTI	0	1
Cardio-vascular	0	0
Mesh infection	0	0
Recurrence	1	0

*N* means absolute number, *SSI* refers to surgical site infection, *UTI* refers to urinary tract infection.

#### CCS scores

The CCS scores are shown in Tables 4 and 5. The CCS scores in TVS group were significantly lower than those in IPOM plus group at one week, one month, and six months after surgery. The numbers of readmission were 0 both in TVS group and IPOM plus group as shown in Table 2.

**Table 4:** Quality of life scores using the Carolina Comfort Scale.

Variable	Symptomatic at 1 week			Symptomatic at 1 month			Symptomatic at 6 months		
	TVS	IPOM plus	*P*	TVS	IPOM plus	*P*	TVS	IPOM plus	*P*
CCS total scores	3.74 ± 1.69	7.55 ± 1.03	< 0.01	0.26 ± 0.86	3.61 ± 1.37	< 0.01	0.15 ± 0.56	1.16 ± 1.65	0.02
Pain	1.62 ± 0.74	3.24 ± 0.43	< 0.01	0.09 ± 0.29	1.32 ± 0.47	< 0.01	0	0.32 ± 0.57	< 0.01
Sensation of mesh	1.06 ± 0.81	2.53 ± 0.73	< 0.01	0.09 ± 0.29	1.32 ± 0.66	< 0.01	0	0.50 ± 0.73	< 0.01
Movement limitation	1.06 ± 0.60	1.79 ± 0.41	< 0.01	0.09 ± 0.29	0.97 ± 0.64	< 0.01	0	0.34 ± 0.63	0.02

CCS is the abbreviation for Carolina’s Comfort Scale.

**Table 5:** Quality of life survey information with the Carolina Comfort Scale.

Variable	Symptomatic at 1 week		Symptomatic at 1 month		Symptomatic at 6 months	
	TVS	IPOM	TVS	IPOM	TVS	IPOM
CCS total (*N*)	20	38	3	29	0	8
Pain (*N*)	18	38	0	12	0	2
Sensation of mesh (*N*)	15	33	0	16	0	5
Movement limitation (*N*)	6	30	0	7	0	3

*N* means the absolute number, CCS is the abbreviation for Carolina’s Comfort Scale.

## Discussion

Recently, several techniques and mesh positioning planes have been developed for incisional hernia, thus, it might be very challenging to choose what to do during ventral hernia surgery^12^. Two of the most popular minimally invasive methods for ventral hernia nowadays are the IPOM plus and e-TEP procedures. Compared to open mesh repair, IPOM plus repair had less infections and wound-healing problems^13^. However, IPOM plus is linked to a higher risk of bowel lesion, adhesive small bowel obstruction (ASBO), intestinal erosion, and greater morbidity following reoperation, with a 21% risk increase for visceral injury^14^. In the e-TEP technique, the benefits of the retromuscular mesh position and the minimally invasive nature of the operation are combined, which can reduce some complications by keeping foreign material out of the abdominal cavity^15^, but break the posterior sheath of retromuscular by the technique of Crossover and TAR. However, the TVS approach would separate the peritoneum only and does not break the structure of abdominal wall. To some extent, TVS could get around them by positioning the preperitoneal mesh correctly to prevent foreign bodies from entering the abdominal cavity. If the prolonged duration of surgery is not taken into account, TVS may be the best choice not only because it maintains the integrity and functionality of abdominal wall as much as possible, but also it has the advantages of the risk for complications, quality of life, postoperative length of stay and cost-effective. The results obtained will be analyzed in detail below.

According to our data, there was no difference in the risk of complications between TVS group and IPOM plus group. There is little literature on TVS technology. As the most comparable technology to TVS, we searched some literatures about e-TEP. The available data on e-TEP was deficient and there were few multicenter reports^5,7^, not mention to TVS. Currently, only a few retrospective and prospective articles compared e-TEP to IPOM^16–18^. Belyansky reported that there were 79 samples with a mean defect area of 132 cm^2^. Only three postoperative complications, including two seromas and one dehiscence at the port site without SSI, were reported, and the median duration of stay was less than two days. And the recurrence rate is 1.3%^5^. Tang reported 153 cases. The median operative time was 135 min and no severe intraoperative complications occurred. Two patients experienced extraperitoneal bleeding, which was managed by nonsurgical means. There was only one umbilical hernia recurrence. And 5.23% and 3.07% of patients had seroma and chronic pain, respectively^8^. Yeow reported a systematic review and meta-analysis ^19^. A total of 11 studies (2320 patients) were identified. They supposed that the patients who undergone IPOM had a numerically higher risk of SSI compared to patients who undergone e-TEP, but this did not reach statistical significance. There were no significant difference between patients who received intraperitoneal versus extraperitoneal mesh for outcomes of SSI, seroma, hematoma, readmission rates, and recurrence rates. The above studies reported low risks in both IPOM and e-TEP approaches. According to the IPOM experience^20–22^, the option of implanting a conventional macro-porous mesh is ensured by the closure of the defect, linea alba, and posterior rectus sheath, which can reduce the risk of seroma formation, recurrence, eventration, and pseudorecurrence. In all the cases, we routinely closed the fascial defect. This might explain why there were such few seroma and hematoma cases in our study. Therefore, the results of our study were in consistent with the existed studies in the performances of the complications. It is also worth mentioning that the Table 2 shows that the mesh area in TVS group is small than that of IPOM group. Most of this is due to the difficulty in separating the preperitoneal space. But long-term follow-up is necessary to determine whether to increase the recurrence rate.

There were big differences between the quality of life of laparoscopic IPOM plus and TVS. The quality of life was evaluated based on the CCS scores, including pain, mesh sensation, and mobility limitation as three components. There were few studies comparing the quality of life of IPOM plus group and TVS group. Based on our comparative results, we found that TVS could improve the quality of life of patients in the short term within six months. For TVS, the preperitoneal approach provides the possibility for proper position of meshes, sandwiched between the posterior fascia and peritoneum without any fixation. Maybe lack of fixation can be a reason for reduced postoperative pain, mesh sensation and mobility limitation^16^. Contrary to the majority of minimally invasive procedures, IPOM does not always result in pain relief or an improvement in quality of life^3^. Some research revealed the connection between aggressive double crown fixation and/or transfascial sutures and postoperative pain^23^. Following IPOM repair, early postoperative pain is prevalent. The reported incidence ranges from 20 to 40%, and this pain, which can last up to 6 weeks to 3 months, makes it difficult for patients to recover and resume their normal activities quickly^18^.

The higher quality of life scores was also in consistent with the longer postoperative length of stay and higher medical expenses in the IPOM group. The TVS approach offers a higher level of cost-effectiveness. The postoperative length of stay and medical expenses in the TVS group are significantly lower than those in the IPOM plus group. The cost of medical care is drastically lowered because of the no use of the expensive composite mesh and fixation tacker in the TVS group, which further mitigates the complications and reduces the postoperative length of stay.

Despite the above-mentioned advantages, the costs had to be paid with the prolonged operative time and ergonomic problems for the surgical team. For TVS group, we report mean operative time of 213.4 min, which is significantly longer than that of IPOM plus group^24^. The surgical procedure is considerably more complex. We have summarized the following practical experience. The learning curve for TVS approach may be steep, requiring advanced laparoscopic skills. The surgeon should make preoperative surgical planning for trocar layout for convenient operation according to the size and site of defect and previous surgical scars. They may benefit from reasonable trocar placement in terms of technical and ergonomic benefits, such as decreased physical stress and strain while improved visualization and access to target tissues. The main issue is that preventing abdominal wall bulging and recurrence requires firm closure of the anterior defect. Although our limited experience, we consider that the TVS approach can be also applied for some simple cases, such as many types of small and medium-sized ventral and incisional hernias. However, given the limitations of the workspace and the rigid, stiff instruments, we hypothesized that the TVS technique is not adequate to manage large and complex defects^25–28^.

The study's limitations include the limited sample size and the non-multicenter study. There is not enough data from the follow-up phase to draw any judgments about the long-term complications. The study is retrospective in design. To demonstrate the method's potential advantages, solid proof and randomized control trial are required.

## Conclusions

The TVS approach, which involves preperitoneal ventral hernia repair, is a viable minimally invasive method. Our findings indicate that both the TVS and IPOM plus techniques yield comparable outcomes in terms of complications on the basis of protecting the abdominal wall structure. TVS approach have better quality of life in the short term within six months, higher cost-effective and shorter postoperative length of stay than those of IPOM plus approach but takes longer operative time. Besides, it's important to consider the long-term advantages of the preperitoneal mesh location in TVS technique over an intraabdominal prosthesis in IPOM plus technique.

### Supplementary Information

Supplementary Information 1.

Supplementary Information 2.

Supplementary Video 1.

## Data Availability

All data generated or analyzed during this study are included in its supplementary information files.
